# Rare Epithelioid Leiomyoma of the Vagina Exhibiting a Pelvic Mass

**DOI:** 10.1155/2017/2190135

**Published:** 2017-02-28

**Authors:** Yuji Tanaka, Mayuko Nagasaka, Mariko Takahashi, Masashi Kobayashi

**Affiliations:** Department of Obstetrics and Gynecology, Kohka Public Hospital, Minakuchichomatsuo 1256, Koka, Shiga 528-0074, Japan

## Abstract

Epithelioid leiomyoma of the vagina is extremely rare. Smooth muscle tumors of the vagina usually present with a submucosal growth pattern or a pedunculated growth pattern from the anterior vaginal wall into the vaginal cavity. Here we report a case of a 43-year-old woman with a solid epithelioid leiomyoma and a palpable mass in the pouch of Douglas. Transvaginal biopsy and angiography showed the epithelioid leiomyoma feeding from the vaginal artery behind the posterior vagina. An abdominal wide excision of the tumor with a partial vaginectomy was performed. Our use of ultrasound-guided needle biopsy and angiography was useful for preoperative diagnosis of a vaginal epithelioid leiomyoma exhibiting a pelvic mass.

## 1. Introduction

**Figure 1:**
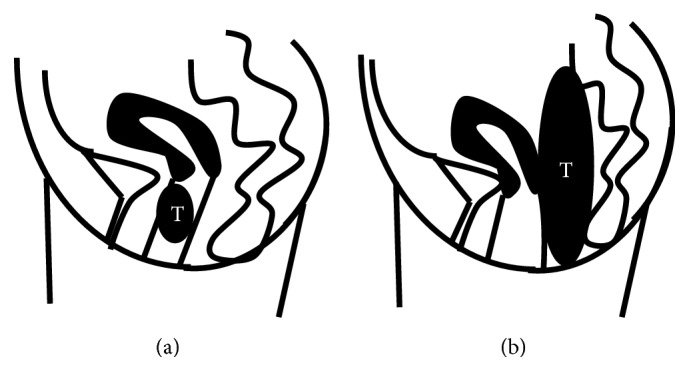
(a) A schematic is shown of the typical location of a smooth muscle tumor (T) of the vagina with a submucosal growth pattern or a pedunculated growth pattern from anterior vaginal wall into the vaginal cavity. (b) In the present case, a vaginal tumor (T) presented a pedunculated growth pattern from the posterior vaginal wall into pelvic cavity, occupying the pelvic cavity.

Epithelioid leiomyomas of the vagina are extremely rare benign smooth muscle tumors. Usually, smooth muscle tumors of the vagina present with a submucosal growth pattern or a pedunculated growth pattern from the anterior vaginal wall into vaginal cavity [1] (Figure 1). Preoperative determination of the primary organ is difficult when a vaginal tumor presents a pedunculated growth pattern into the pelvic area. Typically, for this condition a complete surgical resection is part of a multimodality treatment, and the determination of the primary organ and a diagnosis of benignancy or malignancy are desirable for informing the method of surgical treatment of the tumor. Here, we report a case of epithelioid leiomyoma in an extremely rare location.

## 2. Case Presentation

**Figure 2:**
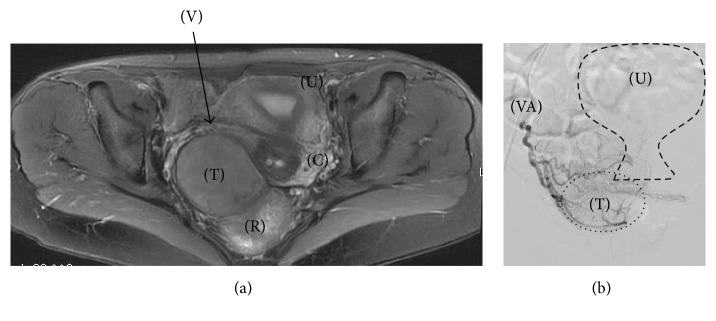
(a) Contrast enhanced MRI radiological analysis. A transverse section shows the tumor (T) anteriorly compressing the uterus corpus (U), cervix (C), and vaginal canal (V), posteriorly compressing the rectum (R). (b) Angiography frontal section image showing that the tumor (T) was nourished from the vaginal artery (VA) and the uterus corpus (U).

**Figure 3:**
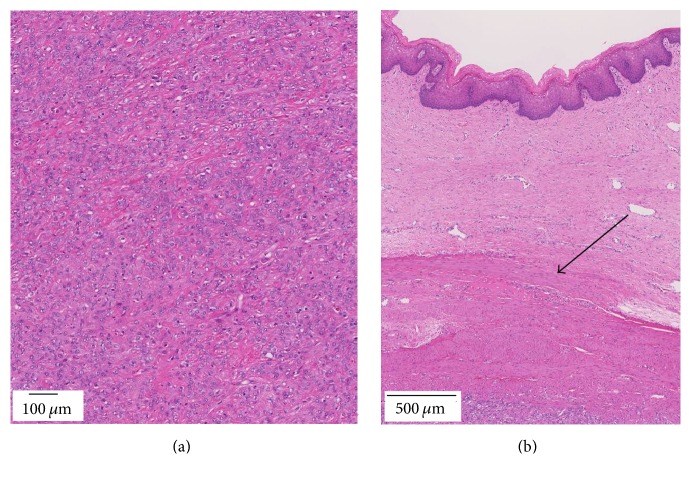
(a) Microscopic findings show the tumor with epithelioid short spindle-cell growth without necrosis. Nuclear pleomorphism is mild. No mitotic activity is present. (b) The tumor involves the vaginal smooth muscle layer and originated (→) from the vaginal wall.

**Figure 4:**
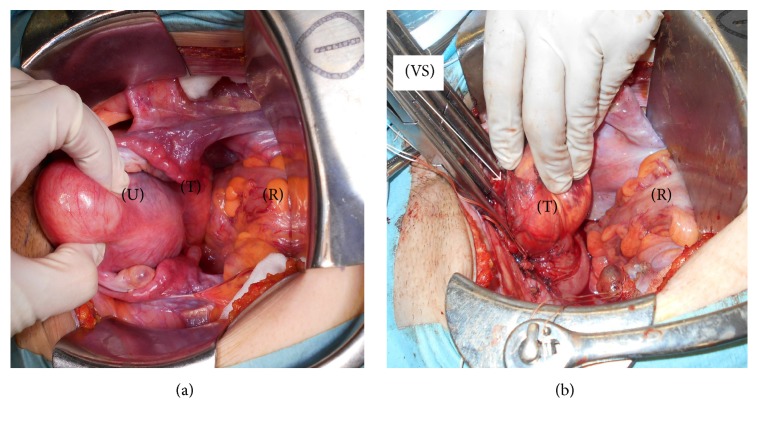
(a) Laparotomy findings. A pelvic tumor (T) was not connected to the uterus (U). (b) Laparotomy findings after an abdominal hysterectomy and a bilateral salpingectomy. A pelvic tumor (T) was connected to the vaginal stump (VS), but not the rectum (R).

**Figure 5:**
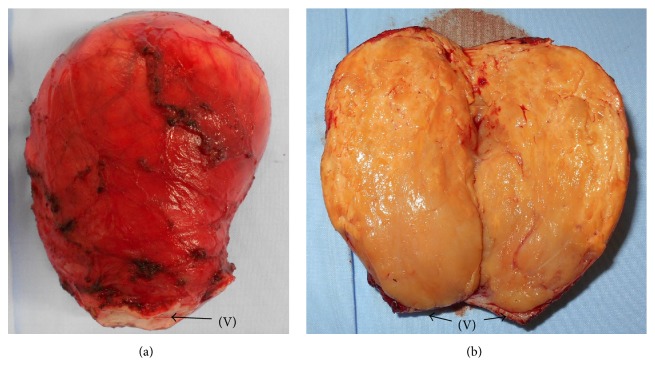
(a) Macroscopic findings of the tumor with connected vaginal (V) tissue. (b) The cut surface of a tumor shows occasional nodules of white fasciculated tissue.

A 43-year-old* primigravida* female was admitted to our hospital for a Pap smear test. As an incidental finding, an ultrasonography and transvaginal physical examination combined with a bimanual rectal exam revealed a solid rubbery mass of approximately 8 × 6 cm between the posterior wall of vagina and anterior wall of the rectum with no obvious limits. Laboratory findings revealed that lactate dehydrogenase, CA-125, carcinoembryonic antigen (CEA), and CA19-9 were within normal limits. Radiological analysis with an echography and contrast enhanced computed tomography (CT) and contrast enhanced magnetic resonance imaging (MRI) revealed multiple leiomyoma in the uterus a solid-cystic mass (8 × 6 cm) in the pelvic area. The mass did not extend from the uterus corpus, but the findings were insufficient to determine its origin (Figure 2). On a diffusion-weighted MRI scan, uterine tumors that were a suspected leiomyoma were hypointense, and a pelvic tumor was hyperintense. Sigmoidoscopy showed a mass pressing the anterior wall of the rectum with no mucosal abnormalities. An ultrasound-guided transvaginal needle biopsy was performed. Microscopically, the tumor showed epithelioid short spindle-cell growth without necrosis. Nuclear pleomorphism was mild, and no mitotic activity was detected (Figure 3). The immunohistochemical staining pattern was partially positive for alpha-smooth muscle actin, desmin, and h-caldesmon. The tumor was negative for pan cytokeratin (AE1/AE3), which excluded epithelium and endothelium differentiation. Melanoma markers melanin A and HMB45 were also negative, which excluded diagnosis of a perivascular epithelioid cell tumor or melanoma. CD34 expression was negative, which excluded solitary fibrous tumor. Estrogen and progesterone receptor were both positive. Ki-67 positive cells were less than 5% of the tumor cells. The pathological diagnosis was an epithelioid leiomyoma. To determine the tumor origin, an angiography was performed, revealing tumor feeding from the vaginal artery, but not the inferior mesenteric artery or the uterine artery (Figure 2). Based on this finding, we suspected that the tumor originated from the vagina. An abdominal wide excision of the tumor with a partial vaginectomy, hysterectomy, and bilateral salpingectomy were performed. Upon laparotomy, the uterine was enlarged with multiple myoma. A pelvic mass was connected to the vagina, but not to the rectum (Figure 4). Firstly, after isolating of ureter, hysterectomy and bilateral salpingectomy were performed. The vagina was transected approximately 1 cm below the cervix. The edges of the vagina were picked up with straight clamps. Secondly, The mass and the connected small posterior vaginal wall measuring 2 × 2 cm were excised en bloc. Thirdly, the edges of the vagina were closed. Macroscopically, the mass measured 8 × 6 cm and had well demarcated borders, and a cut surface revealed occasional nodules of white fasciculated tissue (Figure 5). The microscopic findings were the same as those of the noodle biopsy. The tumor involved the vaginal smooth muscle layer and originated from the vaginal wall (Figure 3). Multiple uterine tumors were diagnosed as a leiomyoma; pathological characteristics of these tumors were different from those of the vaginal tumor. The patient was discharged one week after surgery, and since the time of this report no recurrence has been observed for more than 14 months.

## 3. Discussion

Almost all of the smooth muscle tumors of the gynecological organ that have been reported were in the uterus. Vaginal smooth muscle tumors are rare. There are several histological types of vaginal smooth muscle tumors. Tavassoli and Norris reviewed the pathological features of 60 cases of vaginal smooth muscle tumors and found two tumors that were epithelioid leiomyomas [2]. Only these other two cases of vaginal epithelioid leiomyoma have been reported in the English literature. Epithelioid leiomyoma is more commonly observed in the stomach (59–94% [3–5]), in other gastrointestinal tract regions, the mesentery, and the uterus [3, 4]. However, few cases have been reported for the gynecological organ, 16 cases in the vulva [6], and two cases in the vagina [2]. A summary of our review of the literature on the reports of epithelioid leiomyoma of vagina is presented in Table 1. Our report is the third report in the English literature of an epithelioid leiomyoma of the vagina.

The rectum, vaginal canal, uterine, and the Denonvillier fascia (as it contains smooth muscle cells and blood vessels [7]) can all be possible origins of a pelvic smooth muscle tumor. Smooth muscle tumors of the vagina are usually located in the anterior wall [8] and rarely in the lateral wall [9] and fornix [1]. Usually, smooth muscle tumors of the vagina form well-delineated submucosal anterior vaginal wall masses or are vaginally pedunculated (Figure 1). Tavassoli and Norris reported the possibility that smooth muscle tumors of the vagina can also be multiple [2]. In the present rare case, a pedunculated vaginal tumor presented from the anterior vaginal wall, occupying the pelvic cavity; thus, the preoperative determination of the primary organ was difficult (Figure 1).

Radiologic (CT or MRI) analysis is mandatory to evaluate a vaginal mass and its relationship to adjacent structures [10]. Although radiological analysis is important, no test provides sufficient sensitivity or specificity to conclusively rule out malignancy, as this determination must be performed based on a histopathological examination [11]. The radiological findings (CT and MRI) of the present case were insufficient to determine the tumor origin.

Determination of the primary organ and a benign or malignant diagnosis are desirable to inform the choice of the surgical approach. In our case, the pathological findings were based on an ultrasound-guided transvaginal needle biopsy, which can be safely performed. In addition, we could determine the tumor origin using an angiography.

Histopathologically, benign and malignant vaginal smooth muscle tumors are difficult to distinguish. The criteria used for diagnosing uterine and soft tissue smooth muscle tumors are markedly different. Tavassoli and Norris established diagnostic criteria for smooth muscle tumors of the vagina that indicate that any tumor with greater than 5 mitoses per 10 high power fields and moderate to marked atypism should be considered as a leiomyosarcoma [2]. When the morphological diagnosis is difficult, biomarkers, such as p16, p53, and ki-67, can be used to predict the behavior of a tumor [11].

Complete excision of a tumor with its capsule and a surrounding rim of normal tissue are recommended for patients with a vaginal leiomyoma [12]. Dhaliwal et al. have specifically recommended the removal of the tumor en bloc to avoid any possible recurrence [13]. Although morcellation of the tumor might allow for an easier surgery approach through a vaginal or abdominal route, tumor recurrence is of concern [8].

In Zhao's series, most epithelioid vulvar leiomyoma patients did not show recurrence at 2 years after excision, but three patients showed recurrence 11 months and 1 year and 10 years later [6]. Thus, long-term follow-up is encouraged for patients with vaginal leiomyoma.

In conclusion, angiography is useful for determining the origin of a pelvic mass. An ultrasound-guided needle biopsy is a useful and safe method for preoperative diagnosis of an epithelioid leiomyoma of the vagina.

## Figures and Tables

**Table 1:** A summary of the epithelioid leiomyoma cases of nonuterine gynecological origin.

First author, year	Age	Size (cm)	Outcome
Our case, 2017	43	8	No recurrence in 14 months
Tavassoli, 1979	34	1.2	No recurrence in 14 years
Tavassoli, 1979	—	—	—
